# Intrathecal IgG Synthesis: A Resistant and Valuable Target for Future Multiple Sclerosis Treatments

**DOI:** 10.1155/2015/296184

**Published:** 2015-01-08

**Authors:** Mickael Bonnan

**Affiliations:** Service de Neurologie, Hôpital F. Mitterrand, 4 boulevard Hauterive, 64046 Pau, France

## Abstract

Intrathecal IgG synthesis is a key biological feature of multiple sclerosis (MS). When acquired early, it persists over time. A growing body of evidence suggests that intrathecal Ig-secreting cells may be pathogenic either by a direct action of toxic IgG or by locally secreting bystander toxic products. Intrathecal IgG synthesis depends on the presence of CNS lymphoid organs, which are strongly linked at anatomical level to cortical subpial lesions and at clinical level to the impairment slope in progressive MS. As a consequence, targeting CNS lymphoid lesions could be a valuable new target in MS, especially during the progressive phase. As intrathecal IgGs are end-products of these lymphoid lesions, intrathecal IgG synthesis may be considered as a specific marker of the persistence of these inflammatory lesions. Here we review the effect upon intrathecal IgG synthesis of all drugs ever used in MS. Except for steroids, all these therapeutic strategies, including rituximab, failed to decrease intrathecal IgG synthesis, with the exception of a questionable incomplete action of natalizumab. Thus, IgG synthesis is a robust marker of persistent intrathecal inflammation and its complete normalization should be one of the goals in future therapeutic strategies.

## 1. Introduction

Intrathecal IgG synthesis is a key biological feature of multiple sclerosis (MS). A growing body of evidence suggests that intrathecal IgG secreting cells may be pathogenic either by a direct action of toxic IgG or by locally secreting bystander toxic products of B-cells (review in [1]).

We first provide a brief introduction to the synthesis pathway of intrathecal IgG in the context of CNS lymphoid organs. As intrathecal IgGs are end-products of these lymphoid infiltrates, intrathecal IgG synthesis may be considered as a specific marker of the persistence of these inflammatory lesions. Here we review the consequences on intrathecal IgG synthesis of all drugs given in the past in MS. We make special mention of rituximab and natalizumab owing to their paradoxical action on intrathecal IgG synthesis.

## 2. Pathway of Intrathecal IgG Synthesis

Multiple sclerosis (MS) is characterized by intrathecal IgG synthesis that occurs as a very early event and is the most robust diagnostic biological criterion of the disease. Either an elevated IgG index or oligoclonal bands (OCB) are found positive in more than 95% MS patients [2, 3]. In the few patients (<0–5%) lacking intrathecal synthesis, a repeated cerebrospinal fluid (CSF) exam is often positive [4–7] and intrathecal secretion is demonstrated by many other techniques such as MRZ reaction, high CSF IgA synthesis [8, 9], oligoclonal free *κ* light-chains [10], or clonal *V*_*H*_ and CDR rearrangements [11, 12], suggesting that OCB and IgG index tests are insufficiently sensitive.* Once acquired, intrathecal synthesis persists mostly unchanged over time [13–17] and never disappears.* Moreover, the intimate affinity maturation of IgG and the peptidic targets of OCB IgG persist over time [2, 18, 19]. As a consequence, each patient has a unique pattern “OCB fingerprint” of CSF immunoglobulins [20, 21]. This biological signature may be scored according to the typical positions of mutational replacements (hotspots) on IgG and can be used as a composite signature* Z*-score, which is highly predictive of the conversion of clinically isolated syndromes (CIS) to clinically defined MS [22, 23]. These hotspot codons reside in the complementary determining region (CDR) where they are predicted to have contact with the (unknown) antigen(s).

A growing set of evidences points to a central role of compartmentalized lymphoid tissue (tertiary lymphoid organs, TLO) in the formation and maintenance of intrathecal IgG synthesis ([24, 25], review in [26–28]). The main somatic hypermutations found in IgG are to be found in the CDR [12], which is targeted by the enzyme activation-induced cytidine deaminase (AICD) that is specifically expressed by B-cells in the context of lymphoid organs. The complex process of IgG affinity maturation also requires dendritic cells as professional antigen-presenting cells; cognate maturation of B-cells in collaboration with T-cells; clonal proliferation and selection in local germinal centers, provided by the TLO structure. Moreover, indirect evidence of intrathecal lymphoid structures is provided by deep analysis of the clonal lineage of IgG and B-cells inside and outside the CNS. CSF IgG, plasmablasts, and plasma cells are expanded from a few single ancestors and are clonally related. Although present on both sides of the blood-brain-barrier (BBB), clonal B-cell and T-cell lineages are mostly confined to the CNS, sometimes “private” to brain regions, indicating a mainly local continuous affinity maturation inside the CNS [29–33]. In animal models, the TLO correlates with epitope spreading in T-cells [34].

Furthermore, both intrathecally synthesized IgG and the underlying lymphoid organs are potentially involved in the pathophysiology of cortical lesions (review in [1, 35]). Although no specific target has yet emerged, converging evidence indicates that IgG may directly target CNS structures [36–40]. Inflammatory cells may also be toxic owing to IgG-independent mechanisms [41–44]—TNF*α*, lymphotoxin, and IFN*γ* being good candidates [45] and B-cells their potential source [46]. Finally, the meningeal lymphoid tissue observed in MS patients is spatially correlated to type III cortical lesions [25, 47, 48]. These cortical lesions represent half of the cortical lesions, cover up to 40–60% of the cortical ribbon in progressive MS, and are associated with a major neuronal loss [49–51]. Lastly, both cortical lesions and TLO strongly correlate with clinical impairment [25, 35, 47, 52].

*In conclusion, intrathecal synthesis is an early-occurring event in the course of MS, which, once acquired, persists essentially unchanged throughout life and may be involved in the pathogenesis of progressive MS. Since cortical subpial lesions, TLO, intrathecal IgG synthesis, and impairment are intimately linked, it might be valuable to target B-cells situated in the TLO and to evaluate the effect in terms of their final product, that is, intrathecal IgG synthesis.*

## 3. Technical Limitations in CSF IgG Measurement Leading to Underestimation of Ig Synthesis

Techniques and norms have been developed in order to help clinicians for the diagnosis of MS [53]. For example, unique CSF BOC are discarded due to low specificity and the cut-off for CSF IgG is high [54]. We propose here shifting from the usual point of view to examining techniques used to demonstrate intrathecal synthesis as low as appears in known MS patients.

CSF is actively secreted by choroid plexus and drains into the venous flow. Blood proteins passively enter the CSF through natural leakages of BBB. Albumin is not secreted in the CSF and comes exclusively from blood. A direct measurement of the IgG synthesis rate would be useful but unfortunately the existing formulae provide limited information.

The de novo CNS IgG synthesis rate (Tourtellotte's formula) is calculated as

(1) IgGSYN=IgGCSF−IgGserum369−AlbCSF−Albserum230  ×IgGserumAlbserum0.43×5,

based on isotope studies in MS patients [55]. Normal values are lower than 3.3 mg/day and the median value in MS patients is 29 mg/day (range 0 to 207 mg/day) [55]. The CSF synthesis rate (5 times a day) is highly variable and the assumption that it is constant is incorrect. Moreover, inconsistent negative results are sometimes obtained so this formula appears unreliable.

The albumin quotient (or ratio), *Q*_Alb_ = [Alb_CSF_]/[Alb_serum_], is a widely used parameter of BBB dysfunction that increases with its permeability. In the basal state, CSF IgG levels come exclusively from the passive diffusion of blood IgG since the latter are not normally secreted inside the CSF. As a consequence, intrathecally secreted IgG cannot be directly measured and are approximated by calculation using various methods. The IgG quotient, *Q*_IgG_ = [IgG_CSF_]/[IgG_serum_], denotes this proportionality, as does *Q*_Alb_.

The widely used IgG index is a dimensionless formula (*Q*_IgG_/*Q*_Alb_) expressing the relative excess from expected CSF IgG [56, 57]. The normal limit of the IgG index is below 0.7, based on the assumption of a linear relation between *Q*_IgG_ and *Q*_Alb_. However, the work of Reiber et al. largely demonstrated the nonlinearity and age-dependence of this relation [2]. This has two major consequences: (1) a slight fluctuation in IgG or albumin level (either caused by therapeutic or natural fluctuation or by interassay variability) may directly impact the IgG index although intrathecal IgG synthesis remains constant. For example, a 10% decrease in serum IgG is directly reported as a 10% increase in IgG index, and day-to-day 10% variations of IgG index are commonly reported [58, 59]. (2) The IgG index limit is thought to be constant whereas it is not and depends on many factors (age, *Q*_Alb_). It would not be a problem if intrathecal IgG synthesis occurred at a high level, but this is unfortunately not the case and the IgG index is commonly found to be close to the normal limit. As a consequence, minor fluctuations in IgG level may be translated into normal or abnormal IgG index results, although the intrathecal IgG synthesis rate is not really impacted.

The normal upper limit of CSF IgG is better assessed by a hyperbolic discrimination line $\left(Q_{\text{Lim}}=(a/b)\sqrt{Q_{\text{Alb}}^{2}+b^{2}}-c^{2}\right)$, where *a*, *b*, and *c* are constants, taking into account a wide range of normal population, age, and BBB permeability [2]. Displaying *Q*_IgG_ in a hyperbolic diagram, the Reibergram gives a more exact appreciation of intrathecal IgG synthesis. The fraction of intrathecally synthesized IgG (expressed in %) is IgG_IF_ = [1 − *Q*_Lim_/*Q*_IgG_] × 100. The median IgG_IF_ in MS is 43% (max. 86%), meaning that 57% of CSF IgG comes from blood [2]. The intrathecally synthesized IgG concentration (in mg/L) in CSF is IgG_Loc_ = (*Q*_IgG_ − *Q*_Lim_)×[IgG_serum_], with a median value of 26 mg/L (max. 172 mg/L) in MS [2]. *IgG*_*Loc*_ *is the only accurate unbiased calculation available of intrathecally synthesized IgG*, giving quantitative results.

The Reibergram was constructed using *Q*_IgG_ of >4000 control subjects devoid of intrathecal synthesis. The mean normal *Q*_IgG_ is noted by *Q*_mean_, and *Q*_Lim_—which is *Q*_mean_ + 3  SD (standard deviations) for higher specificity—is the higher discriminant line for abnormal *Q*_IgG_. *Q*_Low_ (*Q*_mean_ − 3  SD) is not used. Very low intrathecal synthesis may be displayed below *Q*_Lim_. In MS patients, *Q*_IgG_ is higher than *Q*_Lim_ in about 75% of cases, which is commonly (and erroneously) interpreted as a null synthesis in the other quarter of patients. However, virtually all MS patients are biased to *Q*_IgG_ > *Q*_mean_ and none is lower than *Q*_mean_, whereas, by definition, *Q*_IgG_ with null intrathecal synthesis should be equally distributed (Gaussian) around *Q*_mean_ [2, 59]. Restraining *Q*_Lim_ at *Q*_mean_ + 2  SD, which includes 96% of controls, increases positivity of *Q*_IgG_ in MS by 10% [59]. This bias demonstrates that* the Reibergram still underestimates intrathecal synthesis and is not sensitive enough to differentiate very low and null intrathecal synthesis*. Moreover, the range of IgG_CSF_ concentrations between *Q*_Low_ and *Q*_Lim_ is wide: for common values of 8 g/L of IgG_serum_ at *Q*_Alb_ 10^−3^, the range of IgG_CSF_ from *Q*_Low_ to *Q*_Lim_ is about 24 to 64 mg/L, which is substantial.

Consensual examination of oligoclonal bands (OCB) is based on IgG staining after an isoelectric focusing (IF) run, which is more sensitive than electrophoresis [60]. Intrathecal synthesis is defined by the presence of CSF restricted OCB and is usually positive in >95% of MS patients [60]. OCB are almost always present when *Q*_IgG_ > *Q*_Lim_ but are also commonly present when *Q*_IgG_ < *Q*_Lim_ [53, 60, 61].

However, isolated monoclonal immunoglobulin bands are sometimes found in CSF. However, owing to their lack of specificity [54], ≥2 OCB are required in most studies to define an oligoclonal pattern with an optimal specificity. Numerous bands are usually unambiguously found in MS patients. The main technical problem is the ambiguity of faint OCB, which are hardly distinguishable from an IgG background, suggesting that techniques attenuating the IgG background may increase test sensitivity. Isoelectric focusing with affinity blotting against known antigens overcomes this limitation. In paraneoplastic syndromes devoid of intrathecal synthesis with classical techniques (OCB, IgG index), affinity blotting against Hu, Yo, or GAD antigens unmasks a specific intrathecal synthesis [62–64]. While affinity blotting gives qualitative results, only OCB restricted to CSF or more pronounced in CSF than in serum are discriminant. This technique improves specific antibody detection in CSF, but the specificity of intrathecally synthesized antibodies in MS is still unknown. Nevertheless, the presence of nonspecific intrathecal synthesis against the neurotropic viruses Measles, Rubella, and VZV (MRZ), commonly observed in MS for unclear reasons, appears very promising and gives a unique opportunity of qualitative and quantitative measures. MRZ-specific OCB are present in the CSF of 72% of MS patients who otherwise fail to demonstrate OCB with IEF [65].

Specific antibody levels are more easily studied using the ELISA technique. The specific antibody index (AI) is a value calculated using the ratio of specific antibodies *Q*_Sp ec_ = [Spec_CSF_]/[Spec_serum_] in the formula AI = *Q*_Sp ec_/*Q*_IgG_, where AI values >1.3–1.5 represent an intrathecal synthesis. For example, high AI values are obtained in paraneoplastic syndrome (anti-GAD, -Hu syndromes) in the absence of apparent intrathecal synthesis (negative OCB and *Q*_IgG_ < *Q*_Lim_) [63, 66]. An MRZ pattern, defined by an elevated AI against ≥2 neurotropic viruses, is observed in up to 90% of MS patients [64, 67]. However, the AI is not usually assessed in patients with *Q*_IgG_ < *Q*_Lim_ and negative OCB, because they are taken to be MRZ-negative since AI correlates with *Q*_IgG_ [61]. A systematic assessment for multiple AI (VZV, HSV, CMV, Measles, Rubella, and* Borrelia*) was undertaken in patients without apparent intrathecal synthesis (*Q*_IgG_ < *Q*_Lim_ and negative OCB). All but one of the 21 miscellaneous infectious and autoimmune patients had at least one elevated AI [61]. In MS patients without intrathecal synthesis, up to 47% of patients have ≥1 elevated AI of MRZ pattern [61, 65, 68–70]. Interestingly, AI results are not completely congruent with those obtained by IEF with affinity blotting, increasing* the prevalence of intrathecal synthesis detected by ≥1 technique to 64% in the “CSF negative” subgroup of MS patients* [65]. Acknowledging that MRZ reaction is common but not exclusive, and that reactions against many other viruses have been confirmed in MS (review in [71]), it may be expected that a larger antigenic test panel would improve the frequency of IT synthesis detection [61].

Free light-chains (FLC) are produced in excess by Ig-secreting cells and are eliminated through renal clearance with a short plasma half-life (2–6 hours). Therefore, the very small amounts of FLC detectable in CSF are less contaminated by passive diffusion from blood. FLC assays in CSF and serum have been tested with various modes of calculation: absolute CSF concentration, *κ*/*λ* ratio, and FLC index. FLC sensitivity seems to be near 100% but the (expected) specificity lower than OCB makes FLC less useful in diagnostic-purposed clinical routine [72, 73]. However, FLC offers a simple and sensitive quantitative parameter, which appears suitable for monitoring intrathecal synthesis.

To our knowledge, no studies combining every technique in MS patients, especially those with “negative CSF,” have been undertaken to date. Such studies performed with the more recent and sensitive laboratory methods would be the only definitive way to examine the prevalence of the true absence of intrathecal synthesis in MS, which is definitely less than 5%. Moreover, we are unaware of such complete reports in non-Caucasian descent MS patients where OCB positivity is low [74, 75]. Our short review argues for a probable faint intrathecal synthesis in the rare “CSF negative” patients. Nevertheless,* common techniques (OCB and IgG index) are positive in more than 95% of patients and Ig synthesis never disappears, making intrathecal synthesis the most valuable marker of MS to date.*

Future studies dedicated to intrathecal synthesis may combine quantitative techniques (IgG_Loc_ or FLC) and highly sensitive qualitative techniques (OCB or MRZ pattern). Criteria for intrathecal Ig synthesis normalization should be based on simultaneous normalization of all the tests. In view of the fluctuations of intrathecal synthesis in individual patients (up to 30% of IgG_Loc_ [76]), the demonstration of a drug action upon intrathecal synthesis should be statistically demonstrated in groups. Aiming at an intrathecal reset, a null Ig synthesis should be confirmed by multiple techniques.

## 4. None of the Available MS Drugs Deplete Intrathecal Ig Synthesis

### 4.1. Steroids

Various protocols of steroid infusions ranging from IV to intrathecal injections of various steroids have been described in the literature but none of them has demonstrated any sustained clinical success upon impairment [77].

In a series of 101 MS patients, CSF was drawn immediately before and after high-dosage steroid blood infusions (1 g/d/10 d) [78]. The proportion of patients having an elevated IgG index decreased from 93% before steroids to 84%. IgM index positivity in 31% of patients was left unchanged (Table 1). OCB were found in 92% of patients and OCB count decreased from a mean of 5 to 4 in 82% of patients [78]. Improvement was only demonstrated in a questionable subgroup analysis. Comparable results were obtained in smaller series, sometimes showing a disassociation between a decrease in IgG synthesis and the preservation of OCB [79–83]. Multiple steroid dosages and various administration routes (intramuscular, intravenous, and intrathecal) had no effect upon OCB [84]. On the contrary, the intrathecal IgG synthesis rate dramatically decreased in all patients (*n* = 22) and sometimes normalized (8/11 after IM ACTH gel) irrespective of the peripheral mode of steroid administration. However, a rebound occurred in a few months.

Intrathecal injections of steroids significantly but transiently depressed IgG synthesis in only 4 patients out of 7 but never normalized the IgG synthesis rate [84]. Even though steroids decreased the IgG index in most but not all patients, the decrease in CSF IgG synthesis was low and the CSF total protein concentration remained unaffected [79]. In a study including 54 MS patients (either progressive or RR), triamcinolone-acetonide (40–80 mg) was intrathecally infused 3–5 times over a week with a positive clinical outcome measured on clinical parameters, but CSF levels of NFL and S100 proteins showed minor changes and immunological parameters were not available [85].

### 4.2. Irradiation

Plasmablasts but not plasma cells are sensitive to irradiation. After irradiation (600–1800 rads) of the whole cerebrospinal axis of 19 MS patients over 4–17-day periods [81], only a transient drop in IgG synthesis rate was observed in 3/7 patients who received 1800 rads, without any effect upon clinical parameters. A combination of ACTH, brain irradiation, and daily prednisone given in 5 patients seemed to block IgG synthesis over a prolonged period and tended to persist after cessation [86]. However, even though CNS IgG synthesis failed in the normal range, the OCB pattern and free light-chains persisted [86].

### 4.3. Interferon Beta

Immunomodulatory therapies had no effect on CSF free light-chain levels [87]. Weekly *β*-IFN given intrathecally had no effect upon IgG_Loc_ or OCB at 6 months [20]. In the phase III study of IFN-*β*1a, essentially no change occurred for IgG index or OCB in CSF drawn at 104 weeks [88]. This absence of effect upon IgG parameters was explained by the ability of IFN to stimulate in vivo expression of IL4 and secretion of IgG [88], but the absence of action upon plasma cell IgG secretion is more plausible.

### 4.4. Azathioprine

Azathioprine (2–4.5 mg/kg/d) had no effect upon CSF IgG synthesis (IgG index and OCB pattern) after one year of treatment [89, 90]. A decrease in the more elevated IgG indexes was observed but patients received steroids (at an active dose in the control group) and the control group had far lower pretherapy index values [90]. In a different study, quantitative IgG synthesis in patients treated by azathioprine did not differ from the control group with or without steroid treatment [91]. No change in CSF OCB and IgG level occurred in 8 patients treated by plasma exchange given in association with prednisolone and azathioprine [92].

### 4.5. Cytarabine (Intravenous or Intrathecal)

Cytarabine (ara-C) is an antimetabolic agent interfering with DNA synthesis and is used in chemotherapeutic regimens for treating lymphomas. Cytotoxic levels administered in the systemic compartment do not achieve cytotoxic levels in the CSF, and reciprocally [93]. None of the 10 patients given cytarabine either systemically or intrathecally had changes in the number or pattern of CSF OCB. CNS IgG synthesis slightly decreased (about 10%) after systemic infusion during the next month. However, CNS IgG synthesis transiently rose during the week following intrathecal administration, while at the same time CSF floating cells were low, and then all returned to their pretreatment level [93].

### 4.6. Lomustine

Lomustine (CCNU) is an alkylating agent used in neurooncology thanks to its high diffusibility in the CSF (~50% of serum concentration) [94]. Given in a single oral dose at 130 mg/m^2^ in four patients, the IgG synthesis rate decreased by −11% at weeks 2 and 4 and by −20% at week 6 (statistically significant from baseline) [94].

### 4.7. 5-Fluorouracil (5-FU)

5-FU infused daily at 10 mg/kg in 3 patients for 5 days showed no effect upon IT IgG synthesis rate [94].

### 4.8. Methotrexate

No data is available about intrathecal Ig synthesis during methotrexate treatment given via the oral or IV route. Methotrexate is widely used via the CSF route in oncology and appears to be relatively safe. In a series of 121 progressive MS patients, intrathecal MTX (12.5 mg/2 months/8 months) proved to be well tolerated [95]. A minor clinical effect was obtained, but in the absence of a control group these results should be considered with caution. Unfortunately, no data was gathered concerning an eventual modification of the CSF (OCB, cells, cytokines).

### 4.9. Cyclophosphamide

High-dosage cyclophosphamide (Cyc) immunosuppression was used in protocols of autologous stem cell transplantation (see [96]). Cyc had a very minor effect upon the IgG index, interacting with the effect of steroids given as cotreatment [97, 98]. Cyc mainly targets T-cells rather than B-cells, and Ig-secreting cells are considerably increased (×4) in blood after Cyc treatment given without steroids, in association with a small increase (~20%) in Ig levels [99]. CSF IgG levels also increased but no data were given about intrathecal secretion [99]. Since the lipophilic properties of Cyc provide access to the CNS through the BBB, CSF levels of Cyc are in the same range in blood and in CSF [100]. Although never investigated, such a paradoxical action may be expected in CSF, as is the case after intrathecal cytarabine infusion. In five patients receiving a daily IV dose of Cyc (2.5–5 mg/kg) for 10 days, CSF IgG synthesis decreased by −11%, which was not significantly different from baseline [94].

### 4.10. Cyclosporine A (CsA)

CsA specifically decreases the biosynthesis of IgG [101]. CsA crosses the BBB with difficulty since levels of CsA varied between 300 and 500 ng/mL in blood and were under the test threshold (30 ng/mL) in CSF [102]. A two-year regimen of CsA (10 then 22 mg/kg/d) resulted in no change in free light-chains in nine patients [102]. In a two-center cohort including 82 patients, half receiving CsA, the IgG index was significantly decreased as compared to placebo in one center but not in the other one [103].

### 4.11. EBV-Specific Adoptive Immunotherapy

A single patient characterized by a blood CD8 T-cell deficiency and a blood EBV-specific CD8 T-cell deficiency (lower than 10th percentile compared to control patients) carrying HLA-A2 and HLA-B7 (which are restricting elements for several EBV epitopes used in this treatment) received autologous CD8+ T-cells activated against EBV [104]. Blood T-cells were collected from the patient, stimulated by AdE1-LMPpoly and IL2 and returned IV by gradual increasing dosage (from 5 × 10^6^ to 2 × 10^7^ cells). Intrathecal IgG secretion and the IgG index were normalized at month 4 [104]. This single case needs confirmation.

### 4.12. Mitoxantrone

Intrathecal IgG synthesis was assessed with a mean follow-up of <2 years in a series of 22 MS patients treated with mitoxantrone [105]. No significant change in IgG index occurred during the follow-up. A modification in OCB bands was observed in 4/22 MS patients after 2 years of treatment, but changes were a gain of new bands in 2 patients or the loss and subsequent replacement of bands in other 2. These changes did not differ from spontaneously occurring changes in untreated patients [105]. However, CXCL13 levels in the CSF at follow-up dropped to levels comparable to those of controls.

In a series of 28 RR-MS patients treated with the association of mitoxantrone (20 mg) + rituximab (1 g) + methylprednisolone (1 g), the frequency and intensity of OCB remained unchanged at 12 months although CD19+ B-cells were profoundly depleted in the CSF [106].

### 4.13. Alemtuzumab

Alemtuzumab targets CD52, which is largely expressed in most lymphocytes, including B-cells but not plasma cells. Serum Ig levels are mostly unchanged after treatment although B-cells are completely depleted. Paired CSF samples available in 15 patients treated with alemtuzumab demonstrated the persistence of OCB following treatment [107].

### 4.14. Cladribine

Essentially no change in the OCB pattern and CSF IgG synthesis occurred after one year of treatment with cladribine [108].

### 4.15. Daclizumab

Daclizumab is a humanized anti-CD25 antibody with a pleotropic effect: expansion of CD56^bright^ NK cells, inhibition of T-cell activation by dendritic cells, and reduction in lymphoid tissue-inducer cells [109]. Daclizumab has a minimal effect on CSF lymphocyte count [110]. After 65 months of daclizumab therapy, the Ig index decreased by only 13% [111].

### 4.16. Fingolimod

Longitudinal CSF analysis during fingolimod treatment of MS patients showed that the cell count decreased (mean 8.3 to 1.8 cells/*μ*L), while the IgG index, which was elevated in 4/7 patients before, remained elevated in 2/8 afterwards and without any mean significant difference, whereas OCB persisted in all patients during follow-up [112].

### 4.17. Vitamin D

Low vitamin D levels are a risk factor for MS and for an unfavorable course of the disease. The median concentration of 25(OH)D was found to be 0.26 nM in CSF and 61.5 nM in blood [113]. CSF levels of 25(OH)D were lower than the in vitro concentration (250 nM) necessary to affect B-cells [114, 115]. However, in two studies with 40 MS patients in each, neither serum nor intrathecal levels of vitamin D correlated with IgG index or presence of OCB [113, 116]. In a series of 36 MS patients, serum vitamin D correlated negatively with IgG index [117]. No data is available about the effect of vitamin D supplementation on intrathecal synthesis.

### 4.18. Autologous Stem Cell Transplantation

Autologous stem cell transplantation (HSCT) in MS provides a unique opportunity to dissect the remaining intrathecal inflammation. Combined myeloablative drugs completely abate the peripheral immune, nonspecifically targeting normal, autoimmune, and neoplastic components. Although HSCT could be expected to be effective in MS, it often fails to halt clinical progression [118–121], relapses [120, 122], and brain atrophy even in the absence of new inflammatory lesions [96, 121, 123]. In a postmortem study of MS patients treated by allogenic HSCT, acute demyelinating lesions and acute axonal degeneration persisted, [124–126] confirming the persistence of ongoing diffuse inflammation.

Receiver intrathecal inflammatory cells persist in CNS even after being completely abated in the periphery after HSCT. Marker of lymphocytes activation (sCD27) is elevated in CSF and only lowered after HSCT, confirming a persistent lymphocytic infiltration [122]. This process was dissected in a female patient having received bone marrow from a male donor and surviving 20 weeks [127]. A postmortem study based on* in situ* fluorescence hybridization performed for X and Y chromosomes in plaques distinguished male donor cells from female recipient T-cells. Male donor cells constituted only 3–17% of CD45 cells and 10% of CD68 macrophages, although all the blood cells originated from the male donor [127]. CD3+ T-cells from the donor were sparse in the parenchyma [127]. This case shows that most of the CNS inflammatory cell pool, which is not limited to nondividing plasma cells, is composed of resident resistant cells and that it seems poorly replenished from the periphery, at least in this context.

Multiple serum OCB usually develop during the first months following HSCT treatment, indicating the probable recapitulation of the B-cell ontogeny after grafting [128]. CSF OCB are rather stable after HSCT grafting: intrathecal pretreatment OCB mainly persists [121], although enriched by multiple OCB diffused from blood because of the disruption of the BBB [128]. In a study of 20 patients, 9 out of the 12 patients having OCB before HSCT were still positive after, and one OCB-negative patient at baseline gained OCB [96]. In a review of 34 HSCT patients, OCB persisted in 30/34 patients while 2 out of the 10 who were initially OCB-negative became positive [122]. One case of SP-MS treated by a nonmyeloablative conditioning allo-HSCT for follicular lymphoma showed the disappearance of baseline OCB for four years, unlike CSF CXCL13 which changed from undetectable to detectable levels [129]. The failure to cure OCB is linked to the persistence of plasma cells rather than to a longer half-life of Ig in CSF [130] since their CSF clearance rate is extremely high. Immunoablation no more abates total blood Ig, IgG, or IgM against various common targets (i.e., myelin, influenza, and tetanus) [131]. This resistance is probably due to the intrinsic properties of long-lived plasma cells residing in bone marrow survival niches.

**Figure 1:**
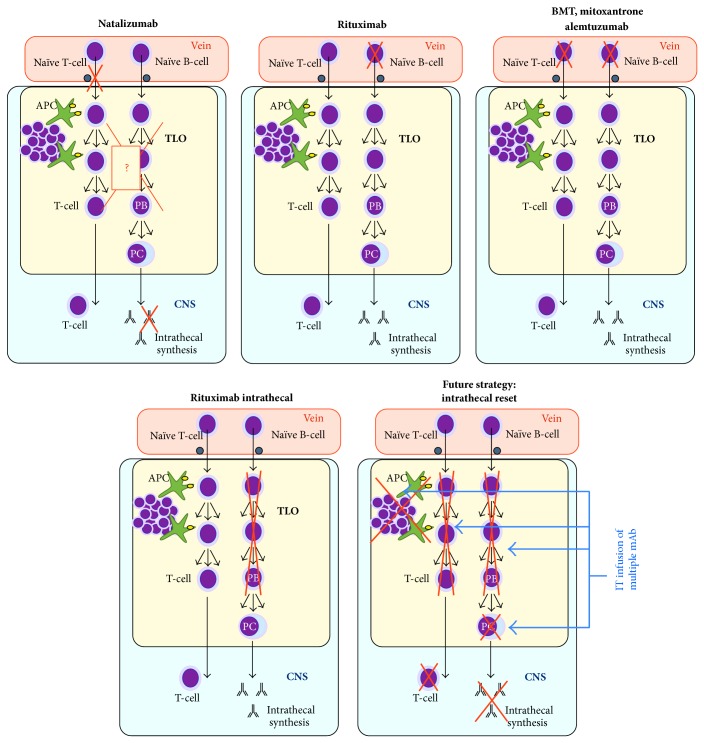
Schematic targets of MS treatments upon CNS compartmentalized inflammation, especially in tertiary lymphoid organs (TLO). Except for natalizumab (owing to unclear mechanisms), none of the treatments targeting blood B-cells have shown any action upon intrathecal IgG synthesis. Preliminary results suggest that rituximab also fails to reduce IgG synthesis. Future treatment strategies might be redirected to reset all the components of intrathecally compartmentalized inflammation. APC: antigen-presenting cells; BMT: bone marrow transplant; PB/PC: plasmablasts/plasma cells; TLO: tertiary lymphoid organs.

*In conclusion, rigorous protocols aiming at the ablation of peripheral lymphocytes have always failed to abate intrathecal IgG synthesis significantly (Figure 1) and as is shown in what follows.*

*Drug Action on Intrathecal IgG Synthesis*

*No Action.* Peripheral administration includes Irradiation, *β*-IFN conventional, Azathioprine, Cytarabine, Lomustine, 5-FU, Methotrexate, Cyclophosphamide, Cyclosporine A, Mitoxantrone, Alemtuzumab, Rituximab, Cladribine, Daclizumab, Fingolimod, Stem cell transplantation. Intrathecal route includes *β*-IFN, Cytarabine, Rituximab.

*Decreasing Ig Synthesis*. Peripheral administration includes steroids, natalizumab (conflicting reports), EBV-specific adoptive immunotherapy (needing confirmation). Intrathecal route includes steroids.

*Normalizing Ig Synthesis.* This was none (only transient effect of steroids).

## 5. Partial Repression of Intrathecal IgG Synthesis by Natalizumab

Natalizumab is a humanized monoclonal antibody directed against VLA-4, preventing leukocyte transmigration to CNS and inducing a sustained decline in all CSF leukocyte subsets up to 6–14 months after cessation of treatment. As soon as the first injection (5 days) of natalizumab and even 6 months after therapy cessation, CSF WBC, CD19+, CD4+ T-cells, CD8+ T-cells, and CD138+ plasma cells are lowered to the same level as those of controls—almost a null count [132]. At 6 months, CD4+ and CD8+ T-cells rose again in only one patient who relapsed [132]. Interestingly, not only T-cells are depleted in MS brain treated with natalizumab but also the same is true for B-cells and dendritic cells, which are usually increased in MS compared to controls [133]. The delayed onset of progressive multifocal leukoencephalopathy (PML) after the first year of natalizumab therapy suggests that the long-term uninterrupted use of natalizumab eventually leads to a reduction in dendritic cells to levels unable to prevent the onset of PML [133]. The number of doses needed to deplete dendritic cells from perivascular spaces, the maximal proportion of depletion that may be expected, and the time to reconstitute the pool after cessation are unknown [133].

In a series of 6 patients treated with natalizumab and positive for OCB before treatment (Table 1), 4/6 patients became OCB-negative (spinal tap controlled by a means of 10 infusions), whereas their IgG index increased in two of the four and dropped in the other two [134]. In a larger series of 76 patients, all the patients had OCB before natalizumab whereas 16% were found to be negative and the proportion of intrathecal synthesized IgG fraction (Reibergram) in the normal range increased from 20% to 45% [135]. In a study involving 24 MS patients, the abnormal IgG index decreased from 67% to 33%, the OCB positive pattern decreased from 92% to 42%, and the mean IgG_Loc_ normalized [136]. Mean IgG_Loc_ was unusually low in this study (mean 0.5 mg/L, max. 2.4 mg/L), which may have biased results toward a high suppressive effect [136]. These results suggest that intrathecal secretion is merely repressed rather than suppressed by natalizumab. In another study including 17 patients, although a minor effect was observed on OCB and IgG index, the changes were not significant at one year [137]. OCB remained detectable in the majority (94%) of 52 patients included in a cross-sectional study [138]. However, a statistically significant but small decrease in IgG_Loc_ and IgG index was observed [138]. AI to MRZ, which was tested in 6 patients, also declined in three of them and remained below the limit in the other three [138].

The repression of IgG synthesis in the intrathecal compartment by natalizumab, which was highly unexpected and paradoxical, deserves three nonmutually exclusive explanations. First, *α*4*β*1-integrin is expressed not only by T-cells but also by B-cells (CD19+ and CD138+) and CD14+ monocytes at higher levels than CD3+ T-cells [139, 140]. As a consequence, B-cell trafficking to the brain is highly impeded by natalizumab and the renewal of the CNS plasmablast pool may also be highly impeded, ultimately leading to the nonrenewal and flush of the terminally differentiated plasma cell pool. Blood retention of lymphocytes after initiation of natalizumab is even disproportionately increased more for CD19+ B-cells than for CD3+ T-cells [140, 141], but this is probably better explained by a purge effect on lymphoid progenitors from bone marrow than by the inhibition of the transmigration of small lymphocytes to the CNS [141]. Secondly, plasma cells survive in niches where they interact with cytokines, and T-cells may also play a supportive survival role among the surrounding cells [45]. Moreover, natalizumab flushes MZ-B-cells from secondary lymphoid organs [141], but whether it has an effect on intrathecal lymphoid tissue cells is unknown. Thirdly, natalizumab inhibits the CNS migration of dendritic cells, which in turn may affect the maintenance of CNS lymphoid tissue [142]. Of note, a significant decrease in IgM (and less significantly in IgG) plasma levels also occurs during natalizumab treatment but is not correlated with treatment duration, suggesting that this drug effectively perturbs the IgG synthesis pathway [138, 143].

Future experiments should examine whether patients who have normalized intrathecal IgG secretion and then discarded natalizumab may regain this secretion. Data obtained from a single patient devoid of OCB under natalizumab and withdrawing treatment for PML showed that OCB shortly returned with a slightly modified pattern [134], whereas a sustained negativation was observed at 6 months in two other patients [135].

## 6. Rituximab Depletes CSF B-Cells without Modifying Intrathecal IgG Synthesis

Rituximab is an IgG1K monoclonal antibody targeting CD20 by multiple and synergistic mechanisms: apoptosis, complement-mediated cell lysis, and antibody-dependent cellular toxicity. All the B-cells expressing high levels of CD20 (CD20^bright^) and a subfraction of CD20+ T-cells are targeted, whereas a minor population of B-cells expressing a lower concentration of CD19 (CD19^dim⁡^) may resist rituximab—even more so at low concentrations—and expand during reconstitution [144]. Plasma cells, which do not express CD20 but secrete high levels of Ig, are fully resistant.

In a single MS case receiving rituximab infusion in blood, CD19+ B-cells were completely depleted from blood and CSF at 8 weeks and 6 months [145]. In another case studied for CSF at 7 time points, CD138+ disappeared after the first infusion until month 7; CD19+ and CD20+ decreased from 3% to 0.25% after the first infusion and then disappeared before month 4 only to reappear briefly at month 5 (0.2–0.4%) and then disappeared again until month 10 [146]. By contrast in blood, CD20+ cells and CD138+ cells, which completely disappeared before the fourth infusion, reappeared at month 10 [146]. In a study of 22 MS patients, CSF was compared before and 24 weeks after IV rituximab [147]. CD19+ and CD3+ cells decreased in CSF but did not completely disappear [147]. The two cytokines CXCL13 and CCL19 decreased in CSF at week 24 compared to baseline, but CXCL10 remained unchanged [147]. In a phase II clinical trial, 16 RR-MS patients were treated by rituximab and were investigated one week prior to and 24 weeks after infusion [76]. In CSF, the CD19+ B-cell count decreased by 90% and the CD3+ T-cell count by 50%. However, the CSF IgG level, IgG index, and OCB number remained essentially unchanged since the overall mean reduction in IgG synthesis of 21% was not statistically significant [76, 147]. In another study (phase II/III trial in PPMS), a minor depletion of CSF CD19+ B-cells was achieved in some patients (1/4) but never to the same extent as in blood [144]. When a second infusion of rituximab was given, the CSF CD19+ cell count no longer dropped either in blood or in CSF [144]. In fact, this failure to lower the intrathecal IgG secretion was predictable from the absence of effect of blood-infused rituximab upon serum IgG and IgA levels, contrary to a minor effect upon IgM levels [76, 148].

Rituximab concentration in CSF does not exceed 0.2% of its concentration in serum, and repetitive infusions in blood fail to increase its CSF concentration [149, 150]. Given the low diffusion of rituximab in CSF and a growing body of evidence demonstrating the safe use of intrathecally infused rituximab, a rationale to infuse intrathecal rituximab in progressive MS recently emerged. Data obtained from a single patient receiving intrathecal rituximab (10 mg each month for 2 months) showed a major effect upon CSF cytokine levels (especially TNF*α*, IL2, IL15, and CXCL10), although intrathecal IgG synthesis was unchanged [151, 152]. These preliminary results deserve further studies and two trials using intrathecal rituximab either alone or associated with intravenous infusion are now recruiting (RIVITaLISE: NCT01212094, ITT-PMS: NCT01719159, in https://www.clinicaltrials.gov/, last access in August 2014). Many aspects of intrathecal rituximab infusion are to be clarified concerning its biological and clinical effects and optimized administration protocols. For example, should the intrathecal route be used alone or associated with blood infusion? Are multiple intrathecal infusions to be scheduled? Do repeated infusions reduce intrathecal IgG synthesis in the long term? Future trials should deal with these pending questions.

## 7. Targeting Intrathecal IgG Synthesis: A Whole New Paradigm in Treating MS

Intrathecal IgG synthesis appears to be partly sensitive to steroids but never abates completely. Since the 70s, authors have hypothesized the existence of two populations of IgG secretory cells that differ in their sensitivity to steroids [80, 153]: one population of B-cells continuously recruited via the attraction of chemotactic factors might be downregulated by steroids, whereas the other population of plasma cells might permanently reside inside the CNS and resist steroids and irradiation [86, 153]. This hypothesis was further supported by the demonstration of a dual population of CNS IgG secreting B-cells: short-lived (days to weeks) fully differentiated plasmablasts retaining their capacity to divide and long-lived (years to decades) plasma cells unable to proliferate.

Some blood-infused treatments have been demonstrated to decrease CSF B-cell levels. For example, B-cell count decreases by 90% after blood infusion of rituximab whereas IgG synthesis remains essentially unchanged. This dissociation suggests that B-cell depletion mainly concerns plasmablasts and CSF floating cells whereas most of the parenchymal residing B-cells (probably plasma cells) remain and secrete IgG. The small decrease in intrathecal IgG synthesis observed after natalizumab is not the consequence of a simple interruption of B-cell traffic into the CNS: traffic is interrupted both after natalizumab and after rituximab infusion, either by traffic blockade or by destruction of blood B-cells. Natalizumab probably provides an additional condition (see above) favoring the attrition of CNS plasmablasts and/or plasma cells. Whatever the exact cause and the duration of the effect are, the few* MS patients losing intrathecal synthesis after steroids or natalizumab provide the proof of concept that intrathecal IgG synthesis is reversible in MS*. Intrathecal infusion of rituximab might overcome the BBB problem and CSF plasmablasts but no plasma cells are expected to be completely eliminated from the CNS. Results of ongoing trials will clarify whether CNS plasmablasts are resistant to rituximab as observed in synovial tertiary lymphoid organs of rheumatoid arthritis and decipher which B-cell subpopulation drives intrathecal IgG synthesis.

Moreover, it has long been thought that the progressive phase of MS is driven by neurodegenerative processes since the compartmentalized inflammation consistently resists immunosuppressive strategies. In our opinion, this point of view cannot be validated until a long-lasting eradication of the CNS immune reaction is obtained. Only after this goal is achieved we will be in a position to assess the possible benefits of immunosuppression in MS [86]. Future therapeutic strategies (Figure 1) may target each component of intrathecally compartmentalized autoimmunity with a “magic bullet” (Ehrlich) associating a few of monoclonal antibodies against key targets, including plasma cells [1]. Depending on the primary antigenic stimulation, the estimated half-life of long-lived plasma cells varies from a decade to one hundred years, and prolonged survival depends on antiapoptotic factors provided by cell niches [154]. Plasma cells expelled from survival niches during competition challenges undergo apoptosis [154, 155]. As a consequence, it cannot be expected that a complete attrition of the terminally differentiated plasma cells residing inside the CNS could be attained in a human life time with road-blocking therapies. Rather, future therapies should also target CNS-resident plasma cells [1].

Intrathecal IgG synthesis is a key characteristic of MS that, once installed at the onset of MS, never fails or abates. Intrathecal IgG synthesis is present in almost every MS patient unlike other CSF markers, which are elevated only in a subset of patients. For example, mean levels of common markers like soluble sCD27 (a marker of T-cell activation), neurofilament light-chain (marker of neurodegeneration), and CXCL13/CXCL10 (attractive cytokines for B-cells) are all elevated in MS series but remain normal in a large proportion of individual MS patients. On the other hand,* IgG synthesis (BOC or IgG index) is always elevated in MS patients and intrathecal IgG level normalization is a goal yet to be attained with treatments. Moreover, a major decrease in intrathecal IgG synthesis, which recapitulates the terminal function of Ig-secreting cells, should be more predictive of an intraparenchymal depletion of the B-cell population. We consider that normalization of intrathecal IgG synthesis, which is easily assessed by spinal tap, could be the main goal in future therapeutic trials targeting intrathecal inflammation.* Future work should attempt to decipher whether the normalization of intrathecal synthesis might be predictive of clearance of CNS inflammation and could be associated with MS remission.

## Figures and Tables

**Table 1:** Posttreatment change in intrathecal synthesis.

References	Drugs	*N*	Time to follow up LP	IgG index^1^	OCB+^2^	IT synthesis^3^
[79]	IV methylpred. (1 g/d/3 d)	7	5 days	IgG 1.7 → 1.1^**^	7/9 → 5/8	Tourt. 26 → 13 mg/L^*^

[78]	IV methylpred. (1 g/d/10 d)	101	10 days	IgG 1.05 → 0.98^**^ IgM 0.13 → 0.12	92% → 82%^**^	IgG_Loc_ 31 → 11 mg/L^**^ (85% → 70%^**^) IgM_Loc_ 1.0 → 0.4 mg/L^**^ (32% → 27%^**^)

[94]	CCNU	4	10 weeks	N/A	N/A	Tourt. Baseline: 59.5 ± 34.8 mg/d Weeks 1-2: −6.3 ± 5.3 mg/d Weeks 3–5: −6.8 ± 8.1 mg/d Weeks 6–10: −11.8 ± 7.5 mg/d^*^

[134]	Natalizumab	6	10 months	(2/4 → 1/6)	(6/6 → 2/6)	N/A

[136]	Natalizumab	24	24 months	0.88 → 0.64^**^ 67% → 33%^**^	92% → 42%^**^	IgG_Loc_ 0.52 → 0.0 mg/L

[137]	Natalizumab	24	60 weeks	Unchanged	(17/17 → 16/17)	N/A

[138]	Natalizumab	59	30 months	Decreased (−0.18)^*^	N/A → 94%	IgG_Loc_ −12.1 mg/L^**^ IgM_Loc_ −0.2 mg/L^**^

[135]	Natalizumab	73	30 months	N/A	100% → 84%^**^	80% → 55%^**^

[76]	IV rituximab	13	24 weeks	Unchanged	Unchanged	Unchanged

[152]	IT rituximab 10 mg/m/2 months	1	2 months	IgG 4.02 → 3.86	Unchanged	N/A

Unless explicitly mentioned, no change is statistically significant. ^1^Mean IgG index is provided when available: elsewhere abnormal IgG indexes (>0.7) are provided as number or per cent of patients. ^2^OCB (IEF) are described as number or per cent of patients. Owing to subjectivity in counting, changes in mean OCB number are not taken into account. ^3^IT synthesis is assessed when *Q*_IgG_ > *Q*_Lim_. Proportion of abnormal synthesis, IgG_Loc_, or mean changes of IgG_Loc_ are reported. N/A: not assessed. Follow-up results without statistically significant changes are categorized as “unchanged.” Since our objective was an eventual normalization of intrathecal synthesis, we did not report synthesis fluctuations when they were not associated with at least normalization of results in some patients. For example, in [76], IgG synthesis rate decreased by 30% in 6 while increasing by 30% in 3 patients, but none achieved normal synthesis. ^*^ *P* < 0.05; ^**^ *P* < 0.01.
